# The continuous low-pressure saline perfusion method of endoscopic submucosal dissection for the circumferential tumor in the remnant rectum after the Hartmann’s procedure

**DOI:** 10.1016/j.vgie.2025.11.004

**Published:** 2025-11-19

**Authors:** Kosei Hashimoto, Tatsuya Yamashita, Kazunori Ogawa, Hidekazu Kurata, Hirotsugu Sakamoto, Edward J. Despott, Yoshikazu Hayashi, Hironori Yamamoto

**Affiliations:** 1 Division of Gastroenterology, Department of Medicine, Jichi Medical University, Shimotsuke, Tochigi, Japan; 2 Division of Gastroenterology, Tochigi Medical Center, Shimotsuga, Tochigi, Japan; 3 Department of Endoscopic Research and International Education Funded by FUJIFILM Medical Co, Ltd, Jichi Medical University, Shimotsuke, Tochigi, Japan; 4 Royal Free Unit for Endoscopy, The Royal Free Hospital and University College London (UCL) Institute for Liver and Digestive Health, Hampstead, London, UK

## Abstract

**Background and Aims:**

Continuous low-pressure saline perfusion in endoscopic submucosal dissection (ESD) combines drainage tubes with saline-immersion therapeutic endoscopy, eliminating bubbles from high-frequency devices, enhancing visualization, and maintaining a deflated lumen. We applied this technique to safely resect a large circumferential lesion in the remnant rectum after the Hartmann’s procedure where the mucosa was very fragile.

**Method:**

An 82-year-old man with a 90-mm circumferential laterally spreading tumor in the Hartmann’s remnant rectum underwent ESD. A nasogastric drainage tube was inserted in an alpha shape, and continuous low-pressure saline perfusion in ESD was performed. The “Asclepius tube” technique also was used to maintain stable drainage and lumen deflation.

**Results:**

The procedure was completed safely within 140 minutes, with excellent visibility and no adverse events noted. Pathology showed an intramucosal, well-differentiated adenocarcinoma with negative margins. A total of 4000 mL of saline was infused, 85% of which was recovered through the drainage tube.

**Conclusions:**

Continuous low-pressure saline perfusion in ESD is a safe and effective technique for large lesions in the Hartmann’s remnant rectum, providing stable lumen collapse, enhanced visibility, and minimal mucosal trauma.

## Introduction

**Figure 1:**
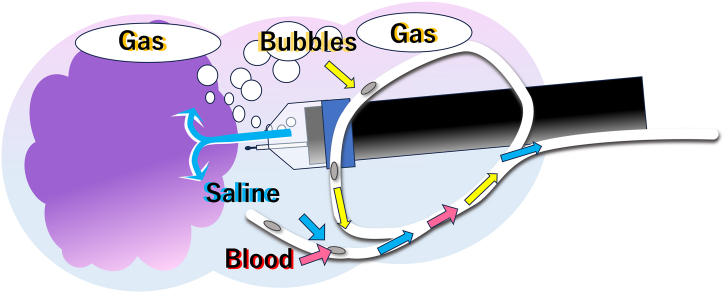
Simply leave the gastric tube open in a cup under the bed, continuously draining the gas, blood, and infused saline passively.

**Figure 2:**
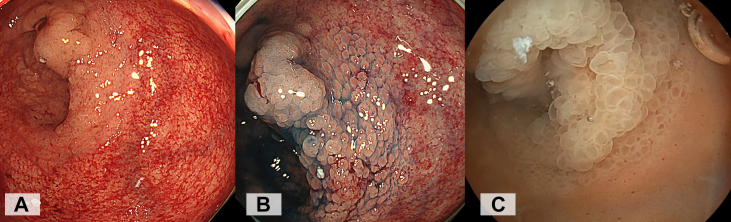
In the remnant rectum following Hartmann's procedure, the fragile mucosa is highly susceptible to damage due to diversion colitis, often exhibiting redness or bleeding upon insufflation during endoscopic observation. As a result, the demarcation line becomes relatively unclear. **A**, White-light image. **B**, Chromoendoscopy with indigo carmine spray. **C**, Underwater conditions without damage of the surrounding mucosa, the demarcation line can be easily identified.

A key technique of the pocket-creation method for endoscopic submucosal dissection (ESD) is maintaining a deflated lumen. This approach facilitates precise dissection by improving maneuverability, reducing tension on the submucosal tissue, and minimizing the risk of perforation.^1^ In 2023, we reported the use of drainage tubes, such as nasogastric tubes or Foley catheters, to achieve continuous and efficient drainage during ESD.^2^, ^3^, ^4^ Combining this method with the saline-immersion therapeutic endoscopy, which has recently gained attention,^5^ has made it possible to perform ESD with continuous saline perfusion, similar to arthroscopic or transurethral bladder surgery.^6^ Continuous saline infusion enhances visibility by eliminating bubbles generated by high-frequency devices, thereby maximizing the benefits of saline-immersion therapeutic endoscopy (Fig. 1). In the stomach and rectum, nearly all of the infused saline is evacuated, minimizing saline volume overload and maintaining the lumen in a consistently collapsed state. We routinely apply this continuous low-pressure saline perfusion technique in nearly all gastric and colonic ESD cases and have found it to be generally effective. One of the key advantages of this method is that continuous saline perfusion within the deflated lumen facilitates the maintenance of a clear visual field. In cases involving the remnant rectum after the Hartmann’s procedure, the fragile mucosa is highly susceptible to damage as the result of diversion colitis, often exhibiting redness or bleeding upon insufflation during endoscopic observation.^7^ As a result, the demarcation line becomes relatively unclear compared with underwater conditions without damage of the surrounding mucosa (Fig. 2). Recognizing this vulnerability, we considered continuous low-pressure saline perfusion ESD as the most suitable approach for treating the circumferential laterally spreading tumor in this anatomical region.

## Case and procedure

**Figure 3:**
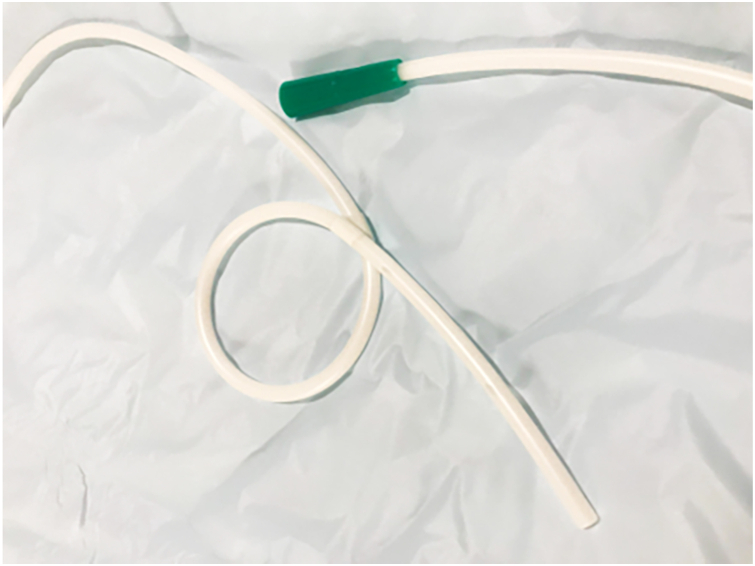
A nasogastric tube (SF-GX1420 14F 125 cm; Terumo, Tokyo, Japan) was shaped like an alpha to prevent it from slipping out of the anus during the endoscopic procedure.

An 82-year-old man with a history of undergoing the Hartmann’s procedure for rectal cancer 12 years previously presented with symptoms of mucus discharge and bleeding from the anus. Endoscopic examination revealed a 90-mm circumferential villous tumor (laterally spreading tumor—nodular-mixed type) in the remnant rectum. The proximal extension of the lesion did not reach the blind-ended anastomosis. To facilitate the procedure, we inserted a nasogastric tube (SF-GX1420 14F 125 cm; Terumo, Tokyo, Japan) in an alpha shape (Fig. 3) to prevent displacement during the endoscopic procedure.^4^ Simply leave the gastric tube open in a cup placed under the bed, allowing the gas, blood, and infused saline to drain continuously and passively.

**Figure 4:**
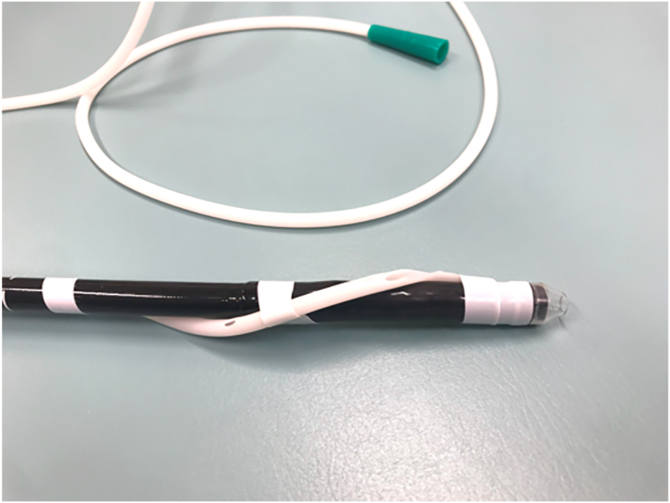
The gastric tube was fixed in a spiral shape to the endoscope using tape. This technique has been referred to as the “Asclepius tube” in our previous articles.

Saline was continuously infused through the accessary channel using a water jet pump (JW-2; Fujifilm, Tokyo, Japan) set to the lowest flow rate. Saline infusion was continuously controlled by an assistant and momentarily paused during instrument exchanges. This low-flow saline infusion, combined with a drainage tube, maintained continuous low-pressure saline perfusion. In the deflated lumen, no mucosal damage occurred, and the demarcation line was sufficiently clear under water conditions; therefore, marking was unnecessary. The procedure was performed using a Tech Knife 1.5 mm (Micro-Tech, Nanjing, China) with the VIO 200 generator (Erbe, Tübingen, Germany) set to endoCUT I (effect 1, duration 4, interval 1) and SWIFT COAG (effect 3.5). After circumferential incision starting from the blind end, submucosal pockets were created from the anal side. Then, the pocket was penetrated to the oral side from the anal side as a tunnel. Because the alpha tube interfered with the circumferential incision line on the anal side, it was removed and subsequently secured to the endoscope using tape to ensure continuous perfusion (Fig. 4). This technique is described as the “Asclepius tube” in our previous article.^8^

Throughout the procedure, the lumen remained deflated, allowing for successful tumor resection without any adverse events within 140 minutes (Video 1, available online at www.videogie.org). The remaining normal mucosa exhibited no signs of damage, such as bleeding or redness, after the procedure. During this procedure, a total of 4000 mL of saline was infused into the rectum, with 85% of it recovered through the drainage tube (the remaining saline was drained directly through the anus or recovered by endoscopic suction). The patient was discharged without any postoperative adverse events such as bleeding, perforation, or infection. The pathologic results revealed a well-differentiated adenocarcinoma with a depth of intramucosal cancer. A follow-up endoscopic examination performed 3 months later revealed stricture within the pouch; however, no clinical problems were observed.

## Conclusions

Continuous low-pressure saline perfusion ESD appears to be an effective approach for treating large lesions on the fragile mucosa of the remnant rectum after the Hartmann’s procedure. This technique provided superior endoscopic visibility and ensured the maintenance of a deflated lumen throughout the procedure, facilitating safe and efficient tumor resection.

## Patient consent

The patient in this article has given written informed consent to this procedure and publication of his case details.

## Disclosure

The following authors disclosed financial relationships: H. Yamamoto: Honoraria, grants, and royalties: Fujifilm. E. J. Despott: Grants and honoraria: Fujifilm, Pentax, Olympus, and Ambu. H. Sakamoto: Research grant and scholarship donation: Fujifilm. All other authors disclosed no financial relationships.

## Supplementary data

Video 1 Continuous low-pressure saline perfusion (CLPSP) technique in endoscopic submucosal dissection (ESD) was applied to safely resect a large circumferential lesion in the remnant rectum after the Hartmann procedure, where the mucosa was very fragile.
